# *In vivo* and *in vitro* evaluation of the cytotoxic effects of Photosan-loaded hollow silica nanoparticles on liver cancer

**DOI:** 10.1186/1556-276X-9-319

**Published:** 2014-06-25

**Authors:** Zhong-Tao Liu, Li Xiong, Zhi-Peng Liu, Xiong-Ying Miao, Liang-Wu Lin, Yu Wen

**Affiliations:** 1 General Surgery Department, Second Xiangya Hospital, Central South University, Changsha 410011, Hunan, P.R., China; 2 State Key Laboratory for Powder Metallurgy, Central South University, Changsha 410083, Hunan, P.R., China

**Keywords:** Nanoscale photosensitizer, Conventional photosensitizer, Photodynamic therapy, Hepatic carcinoma, Cell apoptosis

## Abstract

This study aimed to compare the inhibitory effects of photosensitizers loaded in hollow silica nanoparticles and conventional photosensitizers on HepG2 human hepatoma cell proliferation and determine the underlying mechanisms. Photosensitizers (conventional Photosan-II or nanoscale Photosan-II) were administered to *in vitro* cultured HepG2 hepatoma cells and treated by photodynamic therapy (PDT) with various levels of light exposure. To assess photosensitizers' effects, cell viability was determined by 3-(4, 5-dimethylthiazol-2-yl)-2,5-diphenyltetrazolium bromide (MTT) assay. In addition, apoptotic and necrotic cells were measured by flow cytometry and the expression of caspase-3 and caspase-9 evaluated by western blot. Finally, the *in vivo* effects of nanoscale and conventional photosensitizers on liver cancer were assessed in nude mice. Nanoscale Photosan-II significantly inhibited hepatoma cell viability in a concentration-dependent manner and this effect was more pronounced with high laser doses. Moreover, nanoscale photosensitizers performed better than the conventional ones under the same experimental conditions (*p* < 0.05). Flow cytometry data demonstrated that laser-induced cell death was markedly increased after treatment with nanoscale Photosan-II in comparison with free Photosan-II (*p* < 0.05). Activated caspase-3 and caspase-9 levels were significantly higher in cells treated with Photosan-II loaded in silica nanoparticles than free Photosan-II (*p* < 0.05). Accordingly, treatment with nanoscale photosensitizers resulted in improved outcomes (tumor volume) in a mouse model of liver cancer, in comparison with conventional photosensitizers. Hollow silica nanoparticles containing photosensitizer more efficiently inhibited hepatoma cells than photosensitizer alone, through induction of apoptosis, both *in vivo* and *in vitro*.

## Background

Primary liver cancer is one of the top malignancies around the world with respect to morbidity and mortality [1]. Liver cancer cases reported in China account for 43.7% of people affected by this disease in the world. Still in China, liver cancer is the second most fatal malignancy, accounting for 20.37 deaths per 100,000 individuals [2]. Moreover, liver cancer incidence has steadily increased in recent years and constitutes a serious threat to health in China. The onset of primary liver cancer is relatively asymptomatic, rendering early diagnosis very difficult. In addition, this cancer is difficult to treat because it typically develops from liver cirrhosis and high rates of liver cancer recurrence and metastasis occur even after clinical diagnosis and treatment. Due to various issues, such as lack of specific treatments, limited innovative medications, and dearth of therapeutic options, it is particularly important and urgent to develop new techniques and therapies for diagnosis and treatment of liver cancer [3].

Photodynamic therapy (PDT), a new method developed during the past 2 decades for the treatment of malignant tumors, has shown good therapeutic effects on a variety of solid tumors [4,5]. However, relatively few studies have been conducted to test whether this therapy can be used for hepatic and other intraperitoneal tumors. PDT involves two processes: (1) light sensitivity is achieved by the administration of photosensitizers to patients and (2) light is transmitted through an optical fiber to the region of the body containing the tumor. Irradiation with light of appropriate wavelength will activate the photosensitizer, which transfers energy to oxygen, triggering a series of reactions leading to cell apoptosis or necrosis. Therefore, photosensitizers play a key role in PDT. Conventional PDT efficacy is restricted by insufficient selectivity, low solubility of photosensitizers, and limited penetration depth of the 630-nm laser light, which reduces the PDT efficacy for tumors located in deeper tissues compared with those at the body surface. In order to improve the photodynamic efficacy, a photosensitizer with high permeability and low side effects must be provided [6,7], which allows concentrations to reach the required level for PDT. Recent progress in nanopharmaceutical research has proposed a few methods to tackle these problems [8]. Researchers have developed various types of nanoscale drug carriers to deliver photosensitizers, such as liposomes [4,5], polymer carriers [9], polyoxyethylene cremophor emulsions [10], and microspheres and nanoparticles [11]. Although these carriers improve photosensitizer properties, their use necessarily involves processes to release the loaded drugs that decrease the rate at which tumor cells absorb photosensitizers, extending the period of time required to reach effective concentrations [12]. Therefore, the development of nanocarriers that do not require an extensive process for releasing loaded photosensitizers would greatly enhance photosensitizer effectiveness by shortening this time period. Because nanoparticles are ideal carriers of photosensitizers [13], the use of silica nanoparticles as carriers for photosensitizers is an extremely viable option [14].

In this study, we aimed to compare the inhibitory effects of photosensitizers loaded in hollow silica nanoparticles and conventional photosensitizers on HepG2 human hepatoma cell proliferation and determine the underlying mechanisms *in vitro*. We found that nanoscale photosensitizers were more efficient in HepG2 cell inhibition compared with conventional photosensitizers. In addition, levels of activated caspase-3 and caspase-9 were significantly higher in cells treated with Photosan-II loaded in nanoparticles than free Photosan-II. Finally, treatment with nanoscale photosensitizers increased mouse survival and reduced tumor volume in mice to a greater extent compared with free photosensitizers.

Overall, our data indicate that hollow nanoparticles containing photosensitizers more efficiently inhibit hepatoma cells than free photosensitizers, through induction of apoptosis, both *in vivo* and *in vitro*.

## Methods

### Cell lines

The HepG2 human hepatoma cell line was purchased from the cell center of the Xiangya School of Medicine of Central South University.

### Experimental animals

Specific pathogen-free (SPF)-grade female BALB/c nude mice (26 to 30 days, 18 to 22 g) were obtained from the Shanghai Laboratory Animal Center of the Chinese Academy of Sciences. Mice were housed in SPF-grade animal laboratory of the Second Xiangya Hospital of Central South University in a temperature and humidity controlled room with food and water *ad libitum*. All procedures were approved by the Animal Ethical Committee of Second Xiangya Hospital of Central South University.

### Preparation of nanoscale photosensitizers

Nanoscale photosensitizers were prepared using a one-step wet chemical-based synthesis at room temperature, as previously described [15]. Tetraethyl orthosilicate (TEOS, 99.99%), polyacrylic acid (PAA, M.W = 3,000) were purchased from Aladdin Chemistry Co. Ltd (Shanghai, China). Anhydrous ethanol (99.7%) and ammonia (25% to 28%) were purchased from Sinopharm Chemical Reagent Co. Ltd (China) and Photosan-II (C_34_H_38_N_4_NaO_5_) obtained from Seehof Laboratorium F&E GmbH (Wesselburenerkoog, Germany). The resulted nanoscale photosensitizers (Photosan-II-loaded hollow silica nanospheres, 10 mg/L) showed good sphericity and narrow diameter distribution, ranging from 25 to 90 nm (mean value 37.8 nm). The encapsulation efficiency reached 95%.

### Cell culture and passaging

Cryopreserved HepG2 human hepatoma cells were thawed and cultured in appropriate volume of 10% fetal bovine serum (FBS) in Dulbecco's modified Eagle's medium (DMEM) purchased from Gibco (USA), at 37°C and 5% CO_2_. Cell growth was observed daily, and culture media were changed as needed. Cells grown to logarithmic phase were trypsinized and passaged.

### MTT assay

Two hundred microliters of a 10^5^ cells/mL suspension was seeded into a 96-well plate and cultured as described above. Photosensitizers used were either conventional Photosan or nanoscale Photosan. The following groups were set: (1) blank control, no photosensitizer and no light; (2) photosensitizer-only group, different concentrations of photosensitizers but not light; (3) light-only group, no photosensitizer treatment, exposure to light of different intensities; (4) experimental groups, with cells treated with different concentrations of photosensitizers and exposed to varying intensities of light. Precisely, cells in experimental groups were cultured in the presence of 0, 1.25, 2.5, 5, 10, or 20 mg/L photosensitizer for 1, 2, and 4 h followed by exposure to light at 2.5, 5, or 10 J/cm^2^ and culture for an additional 24 h. Cell inhibition rates were determined after treatment with 3-(4, 5-dimethylthiazol-2-yl)-2,5-diphenyltet-razolium bromide (MTT) obtained from Sigma-Aldrich (St. Louis, MO, USA) as previously described [16]. Each experiment was repeated three times.

### Flow cytometry experiments

Based on the results obtained in MTT assays, four groups shown in Table 1 were analyzed by flow cytometry: Cells were stained using the Annexin-V-FLUOS staining kit purchased from Roche (Nutley, NJ, USA), following the manufacturer's instructions. Briefly, 10^5^ resuspended cells were gently resuspended in 195 μL of Annexin V-FITC binding buffer followed by the addition of 5 μL of Annexin V-FITC and incubation in the dark at room temperature (20°C 25°C) for 10 min. After washing, cells were incubated in binding buffer containing propidium iodide (PI). Annexin V-FITC produced green fluorescence while PI produced red fluorescence. These experiments were repeated three times.

**Table 1:** Four groups with various processing methods

**Group**	**A**	**B**	**C**	**D**
Processing methods	Blank control	PDT treatment and nanoscale Photosan, using optimal parameters for nanoscale Photosan	PDT treatment with conventional Photosan, using optimal parameters for nanoscale Photosan	PDT treatment with conventional Photosan, using optimal parameters for conventional Photosan

### Evaluation of caspase-3 and caspase-9 levels by western blot

Three groups of cells were analyzed: a normal control group (A), a nanoscale photosensitizer group (B), and a conventional photosensitizer group (C). Cells in groups B and C were treated with 5 mg/L photosensitizer and irradiated at 5 J/cm^2^ for 2 h. After treatment, cells were lysed in 500 μL radioimmunoprecipitation assay (RIPA) lysis buffer on ice for 30 min. After centrifugation at 12,000 rpm for 5 min at 4°C, protein concentrations were determined in supernatants using the BCA Protein Assay Kit (Wellbio, China) according to the manufacturer's instructions. Equal amounts of proteins were separated by electrophoresis on a precast 15% polyacrylamide gel and transferred onto polyvinylidene difluoride (PVDF) membranes. After blocking, the membranes were incubated overnight at 4 °C with rabbit anti-human caspase-3/caspase-9 monoclonal antibodies purchased from Boster Biological Engineering Co. (Wuhan, China). After washing, membranes were incubated in horseradish peroxidase (HRP)-labeled secondary antibodies (1:3,000) for 45 to 60 min and detected with an enhanced chemiluminescence (ECL) chromogenic substrate. Images were obtained by autoradiography and scanned for analysis.

### *In vivo* tumor inhibition in a mouse model of liver cancer

Routinely cultured hepatoma cells were collected, washed with PBS, and resuspended in PBS to 2 × 10^6^ cells/mL. A total of 0.2 mL of the prepared cell suspension (4 × 10^5^ HepG2 cells) was injected into the right armpit of each nude mouse and tumor growth observed every other day. Typically, subcutaneous foreleg tumors became visible after 5 to 7 days. At this time, tumor sizes were measured with Vernier calipers, and the long diameter, short diameter, and height of each tumor were recorded. Treatment started when tumor volumes reached approximately 0.5 cm^3^. The mice were randomly divided into the following groups (*n* = 15): normal control animals (neither photosensitizer nor light treatment), and nanoscale photosensitizer and conventional photosensitizer treatment groups. Each animal in the treatment groups received an intraperitoneal injection of 10 mg photosensitizer per kilogram. Four hours later, animals were irradiated with a 63-nm laser (500 mW, 10 min). The subcutaneous xenograft tumors were ellipsoid in shape; thus, tumor volumes were calculated using the equation for ellipsoid volume: *V* = *a* × *b* × *c* × *π* × 4/3 (*a*: long diameter of the tumor; *b*: short diameter of the tumor; *c*: tumor height). After treatment, tumor sizes were measured every other day with Vernier calipers. Tumor dimensions were determined by averaging three repeated measurements. Lag phases in tumor growth before and after treatment and final mouse survival times were recorded.

### Statistical analyses

Statistical analyses were performed using the SPSS statistical software version 12.0 (SPSS Inc., Chicago, IL, USA). All data were expressed as mean ± SD. Comparison of multiple independent samples were performed by one-way analysis of variance (ANOVA) and *p* < 0.05 considered statistically significant.

## Discussion

### Cytotoxic effects of conventional and nanoscale photosensitizer PDT on human hepatoma cells

**Figure 1:**
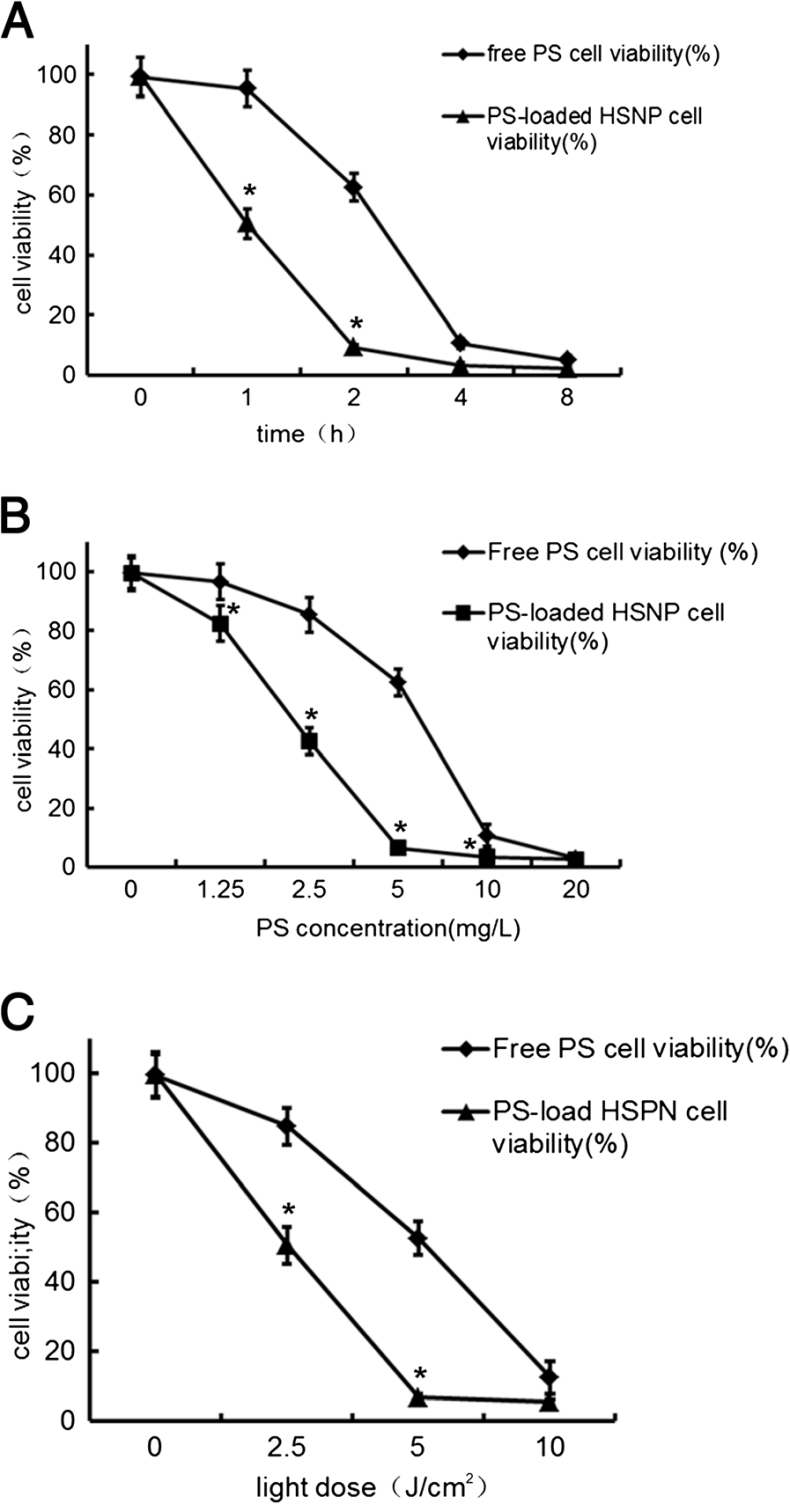
**The impacts of (A) incubation times, (B) Photosan concentrations, and (C) light dose on cytotoxic effects of PDT. (B)** Conventional Photosan and nanoscale Photosan concentrations on cytotoxic effects of PDT. *Significant difference (*P* < 0.05) of cell viability was detected between two groups at the time point.

At fixed photosensitizer concentrations and laser irradiation doses, cell viability was significantly affected by the incubation time. In addition, cell viability was significantly lower in cells subjected to nanoscale photosensitizer-mediated PDTs than in cells treated with conventional photosensitizers. In HepG2 cells treated with 5 mg/L conventional Photosan and irradiated at 10 J/cm^2^, viability declined from 0 to 4 h and remained stable thereafter. In the nanoscale Photosan group, significant differences in cell viability were observed after 1 and 2 h of incubation, whereas cells treated for more than 2 h exhibited no significant differences in cell viability (Figure 1A). According to these data, 4 and 2 h were used in subsequent experiments for conventional and nanoscale photosensitizers, respectively.

At fixed incubation times and laser irradiation doses, cell viability significantly differed with photosensitizer concentrations. In addition, cell viability was significantly lower in cells subjected to nanoscale photosensitizer-mediated PDTs than in cells treated with the conventional. In the conventional Photosan group, cells incubated for 2 h at 10 J/cm^2^ cell showed a gradual decline in viability as Photosan concentrations increased from 0 to 20 mg/L, with significant differences in cell viabilities at different concentrations. At 20 mg/L, no statistically significant differences in cell viability were observed between conventional and nanoscale Photosan treatments. HepG2 cell-treated nanoscale Photosan showed a different pattern: cell viability declined as photosensitizer concentrations increased from 0 to 5 mg/L and stabilize thereafter (Figure 1B). According to these findings, 10 and 5 mg/L were used in subsequent experiments for conventional and nanoscale photosensitizers, respectively.

At fixed photosensitizer incubation times and concentrations, cell viability was significantly affected by light doses. In addition, cell viability was significantly lower in cells subjected to nanoscale photosensitizer-mediated PDTs than in cells treated with the conventional. In the conventional Photosan group, cells incubated for 2 h in the presence of 5 mg/L photosensitizer showed a gradual decline in cell viability as light doses increased from 2.5 to 10 J/cm^2^, with significant differences at different light doses. In cells treated with nanoscale Photosan, significant differences in cell viability were observed between exposure at different light intensities, from 0 to 5 J/cm^2^, with no significant difference in cell viability observed thereafter (Figure 1C). Accordingly, 10 and 5 J/cm^2^ were used in further experiments for conventional and nanoscale photosensitizers, respectively.

### Effects of conventional and nanoscale photosensitizers PDT on human hepatoma cell apoptosis

**Figure 2:**
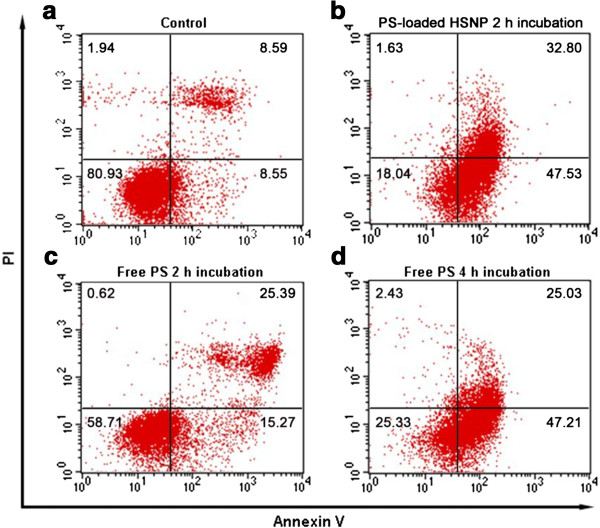
**Flow cytometry analyses of groups A, B, C, and D.** Group **A** cells are the blank control; group **B** cells were treated with 5 mg/L nanoscale Photosan for 2 h at 5 J/cm^2^; group **C**, cells received 5 mg/L conventional Photosan for 2 h at 5 J/cm^2^; group **D** cells were treated with 10 mg/L conventional Photosan for 4 h at 10 J/cm^2^. Lower left quadrants represent normal cells; lower right quadrants are early apoptotic cells; upper right quadrants represent late, dead apoptotic cells; upper left quadrants are mechanically damaged cells. The apoptotic rate was defined as100* (sum of early apoptotic and late apoptotic cells)/total number of cells.

Flow cytometry was used to quantitate apoptosis rates in human hepatoma cells submitted to conventional Photosan-based PDT or nanoscale Photosan-based PDT. Group a cells were the blank control; group b cells were treated with 5 mg/L nanoscale Photosan for 2 h at 5 J/cm^2^; group c cells received 5 mg/L conventional Photosan for 2 h at 5 J/cm^2^; group d cells were treated with 10 mg/L conventional Photosan for 4 h at 10 J/cm^2^. As shown in Figure 2, apoptosis rates for groups a, b, c, and d were 17.14%, 80.33%, 40.66%, and 72.33%, respectively. The treatment groups (groups b, c, and d) significantly differed from the control group a (*P* < 0.05). Total apoptosis rates were similar in groups b and d (*P* > 0.05), and significantly higher in group b compared with group c (*P* < 0.05). Flow cytometry data further confirmed the cytotoxic effects of PDT as detailed above. Under the same experimental conditions, the cytotoxic effects of conventional Photosan-mediated PDT were significantly lower than those of nanoscale Photosan-mediated PDT (*P* < 0.05). In addition, a comparison of conventional Photosan- and nanoscale Photosan-mediated PDT using respective optimal parameters indicated the superiority of nanoscale Photosan in inhibiting cancer cell growth (*P* < 0.05) as shown in Figure 2.

### Caspase-3 and caspase-9 protein levels in hepatoma cells submitted to conventional and nanoscale photosensitizer PDT

**Figure 3:**
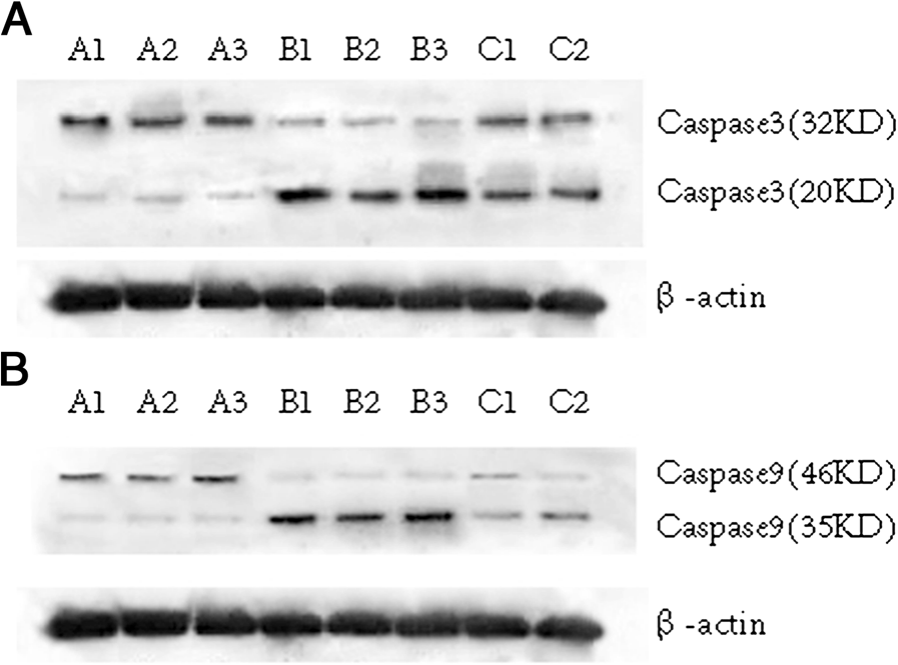
**Active caspase-3 (A) and caspase-9 (B) protein levels in cancer cells after conventional and nanoscale photosensitizer PDT.** A1, A2, and A3: blank control samples; B1, B2, and B3: nanoscale Photosan-treated samples; C1 and C2: Photosan-treated samples.

Western blot data demonstrated that PDT with 5 mg/L photosensitizer for 3 h at 5 J/cm^2^ resulted in higher level of active form of caspase-3 (20 kD) in both nanoscale Photosan and conventional Photosan-treated samples (Figure 3A). Interestingly, caspase-3 levels were significantly higher in nanoscale photosensitizer-treated cells compared with cells treated with conventional photosensitizers (*P* < 0.05). Similar results were obtained for active caspase-9 (Figure 3B).

### Therapeutic effects of conventional photosensitizers and nanoscale photosensitizer PDT on human hepatoma xenografts in nude mice

**Figure 4:**
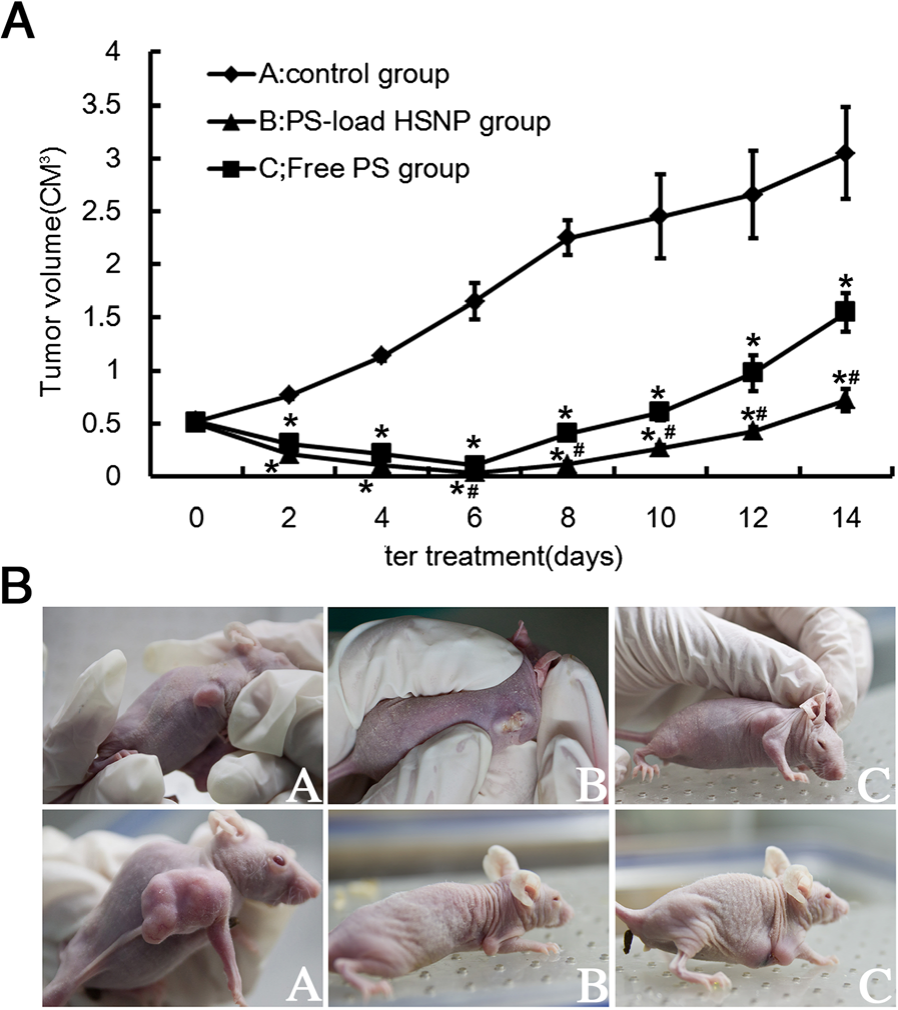
**Tumor volumes after treatments during 14 days (A) and their digital photographs (B).** (A) When tumor volumes reached approximately 0.5 cm^3^, one group of the mice did not receive any treatment (A, Control group) and two groups of the mice received treatment with conventional Photosan (C, Free PS group) and nanoscale photosensitizer (B, PS-load HSNP group), respectively. The tumor sizes were measured in the following 14 days. Significantly different (*P* < 0.05) from group A, ^#^Significantly different (*P* < 0.05) from group C. The digital photograph of the tumor volumes of the three groups before treatment (B) and 14 days after treatment (C). Where, A is the control group; B is PS-load HSNP group and C is the Free PS group.

Table 2 shows the subcutaneous xenograft tumor volumes (cm^3^) in nude mice after various treatments during 14 days. Prior to PDT, no significant differences in tumor volume were observed among groups and before treatment, tumor growth was relatively fast, with tumors reaching 0.5 ± 0.03 cm^3^ 2 weeks after cancer cell injection. In the nanoscale photosensitizer group, significant necrosis in tumor tissues was observed 1 to 2 days after PDT: tumor volumes started to rapidly decrease, and tissue regeneration caused the formation of scabs at the wound surface. After 6 to 8 days, the scab wound surface had been shed, and tumor regrowth was observed. However, tumors were significantly smaller and developed slower in this group compared with control mice and animals treated with conventional Photosan. In conventional Photosan PDT group, the therapeutic effects observed during early stages after PDT treatment were similar to those in the nanoscale Photosan group. However, after the necrotic tissue shedding, scabs formed at wound surfaces and tumors regenerated quickly. Of note, tumor volumes in nanoscale and conventional Photosan groups were significantly smaller than sthose obtained for control animals from 2 days post-treatment throughout the experiment (*P* < 0.05). Tumor volumes were similar in nanoscale and conventional Photosan groups 6 days after treatment; however, after this time point, tumor were significantly smaller in the former group compared with the latter (*p* < 0.05) , as shown in Figure 4A and the digital photograph before treatment (Figure 4B) and 14 days after treatment 4c.

**Table 2:** **Subcutaneous xenograft tumor volumes (cm**^**3**^**) in nude mice**

	**Group A**	**Group B**	**Group C**	***P*** **(A/B)**	***P*** **(A/C)**	***P*** **(B/C)**
1.	15	15	15	-	-	-
2.	0.525 ± 0.019	0.520 ± 0.013	0.527 ± 0.015	0.588	0.876	0.487
3.	0.867 ± 0.031	0.250 ± 0.010*	0.412 ± 0.013*	0.000	0.000	0.856
4.	1.236 ± 0.039	0.112 ± 0.013*	0.217 ± 0.011*	0.000	0.000	0.770
5.	1.750 ± 0.169	0.035 ± 0.014*^#^	0.105 ± 0.038*	0.000	0.000	0.020
6.	2.251 ± 0.162	0.114 ± 0.020*^#^	0.406 ± 0.050*	0.000	0.000	0.001
7.	2.451 ± 0.397	0.266 ± 0.042*^#^	0.608 ± 0.076*	0.000	0.000	0.008
8.	2.657 ± 0.411	0.475 ± 0.058*^#^	1.058 ± 0.170*	0.000	0.000	0.004
9.	3.050 ± 0.438	0.623 ± 0.108*^#^	1.551 ± 0.180*	0.000	0.000	0.000

1. Number of animals; 2. Before treatment; 3. 2 days after treatment; 4. 4 days after treatment; 5. 6 days after treatment; 6. 8 days after treatment; 7. 10 days after treatment; 8. 12 days after treatment; 9. 14 days after treatment; Group A - blank control; Group B - nanoscale Photosan group; Group C - conventional Photosan group; *P*(A/B) - *P* value for comparing group A and group B; *P*(A/C) - P value for comparing group A and group C; *P*(B/C) - *P* value for comparing group B and group C. *Significantly different (*P* < 0.05) from group A, ^#^Significantly different (*P* < 0.05) from group C.

Primary liver cancer (hepatocellular carcinoma) is the most common type of malignant tumor in China. Although surgical excision and liver transplantation therapies can significantly prolong the survival of liver cancer patients, most patients are only diagnosed at later stages and cannot be surgically treated. Therefore, non-surgical approaches play a vital role in the treatment of primary liver cancer; however, non-surgical approaches have generally exhibited extremely limited therapeutic efficacy [17].

PDT is a novel tumor treatment approach developed since the 1980s. The basic principle of PDT is that photosensitizers can be selectively taken up and retained in tumor tissues; thus, the excitation of these photosensitizers by exposure to specific wavelengths of light can generate cytotoxic singlet oxygen atoms and/or oxygen-free radicals that achieve the therapeutic objectives of killing tumor cells, disrupting tumor blood vessels, stimulating immunomodulatory effects in the body, and causing necrosis and shedding among tumor cells [18].

PDT involves lasers and photosensitive drugs (photosensitizers). In particular, the photosensitizers (or their metabolites) under excitation at appropriate wavelengths of light produce photodynamic effects, which can destroy the targeted cells. The introduction, development, and application of PDT have been accompanied by gradual improvement of photosensitizers. However, most photosensitizers discussed in available reports exhibit certain shortcomings mainly related to hydrophobicity or limited solubility in aqueous solutions. This issue causes various deleterious effects that impair the therapeutic value of these photosensitizers, including accumulation in bodily fluids (such as blood), alteration of photosensitizer photochemical properties, and reduction of singlet oxygen production. Recent progress in nano-pharmaceutical research has proposed a few methods to tackle these problems [8]. Silica nanoparticles have drawn increasing attention due to several advantages, including extremely controllable shape and size, good water solubility, stability, and high biocompatibility. More importantly, silica nanoparticles are permeable to small molecules such as singlet oxygen [19,20], the key impact factor in PDT, and the small size of these nanoparticles allows them to permeate through cell membranes [21,22]. Therefore, the use of silica nanoparticles provides clear advantages to overcome conventional nanocarriers that require photosensitizer release processes to occur [23]. Therefore, silica nanoparticles constitute an ideal nanocarrier that can enhance the photodynamic effects of photosensitizers, as shown elsewhere [15].

In *in vitro* experiments, we first used MTT assays to confirm that both conventional Photosan- and nanoscale Photosan-mediated PDT resulted in HepG2 hepatoma cell cytotoxicity. We found that relative to conventional Photosan, nanoscale Photosan was more cytotoxic, required shorter photosensitizer incubation times, and enhanced PDT efficacy. In addition, experiments revealed that nanoscale photosensitizers did not exhibit cytotoxicity. These findings provide a basis for promoting the use of photosensitizers. These findings regarding the relatively higher cytotoxic effects of nanoscale Photosan-mediated PDT were further confirmed by flow cytometry. Under the same experimental parameters, treatment with nanoscale Photosan resulted in significantly improved therapeutic effects than conventional Photosan treatments. Indeed, the use of conventional Photosan at higher concentrations and longer incubation still produced cell death rates significantly lower than that observed in the nanoscale Photosan groups. In addition, we demonstrated that apoptosis is involved in cell death triggered by conventional Photosan and nanoscale Photosan. Interestingly, nanoscale Photosan-mediated PDT produced a higher proportion of apoptotic cells than conventional Photosan.

Furthermore, in *in vivo* experiments using a mouse model liver cancer, changes in tumor volume, tumor growth, and mean mouse survival times in response to treatment were assessed, after treatment with the two photosensitizer types. Our results clearly indicated that significantly better therapeutic efficacy was obtained with nanoscale photosensitizers. These data were in agreement with the *in vitro* findings and provide a solid basis for future clinical trials of photosensitizer carriers.

The mechanisms underlying PDT-induced apoptosis mainly involved two signaling pathways: (1) death receptor-mediated exogenous pathway and (2) mitochondria-mediated endogenous pathway. It is known that activation of the endogenous pathway rather than the exogenous pathway is typically the main cause of PDT-induced apoptosis [24-26]. Cytoplasmic cytochrome C (Cyc) and apoptotic protease-activating factor 1 (Apaf-1) form a heptameric apoptotic complex that binds to, cleaves, and thereby activates the caspase-9 zymogen. Caspase-9 hydrolyzes and activates caspase-3/7, which reaches the same termination point produced by the aforementioned exogenous pathway [27-29]. The death receptor-mediated exogenous (caspase-8) pathway ultimately activates caspase-3 to induce apoptosis. Thus, both pathways eventually induce apoptosis through caspase activation.

Our experiments showed that PDT cells exhibited significantly enhanced levels of active caspase-3 and caspase-9 proteins, which were significantly higher in nanoscale Photosan group compared with conventional Photosan group. These findings indicated that both Photosan-mediated PDT induce tumor cell apoptosis *via* endogenous and exogenous pathways. Relative to conventional photosensitizers, nanoscale photosensitizers exhibited enhanced photochemical efficacy and higher water solubility, and increased effective drug concentrations in tumor tissues. Thanks to these properties, the use of nanoscale enhances the effects of photosensitizer PDT of tumor cells.

## Conclusion

In summary, we performed the *in vivo* and *in vitro* evaluation of the cytotoxic effects of Photosan-loaded hollow silica nanoparticles on liver cancer cells. The results showed that nanoscale photosensitizers were more effective in inhibiting liver cancer cells compared with conventional photosensitizer, both *in vitro* and *in vivo*.

## Competing interests

The authors declare that they have no competing interests.

## Authors’ contributions

LX and Z-PL wrote the paper. L-WL and X-YM revised the paper. L-WL designed and prepared the Photoscan-loaded hollow nanoparticles and YW designed the experiment. Z-TL performed the experiments. All authors read and approved the final manuscript.
